# Development and validation of a machine learning predictive model for perioperative myocardial injury in cardiac surgery with cardiopulmonary bypass

**DOI:** 10.1186/s13019-024-02856-y

**Published:** 2024-06-26

**Authors:** Qian Li, Hong Lv, Yuye Chen, Jingjia Shen, Jia shi, Chenghui Zhou

**Affiliations:** 1 Department of Anesthesiology, State Key Laboratory of Cardiovascular Disease, Fuwai Hospital, National Center for Cardiovascular Diseases, Chinese Academy of Medical Sciences and Peking Union Medical College, Beijing, China; 2 Center for Anesthesiology, Beijing Anzhen Hospital, Capital Medical University, No. 2 Anzhen Rd., Chaoyang District, Beijing, 10029 China

**Keywords:** Perioperative myocardial injury, Cardiac surgery, Machine learning, Logistic regression

## Abstract

**Background:**

Perioperative myocardial injury (PMI) with different cut-off values has showed to be associated with different prognostic effect after cardiac surgery. Machine learning (ML) method has been widely used in perioperative risk predictions during cardiac surgery. However, the utilization of ML in PMI has not been studied yet. Therefore, we sought to develop and validate the performances of ML for PMI with different cut-off values in cardiac surgery with cardiopulmonary bypass (CPB).

**Methods:**

This was a second analysis of a multicenter clinical trial (OPTIMAL) and requirement for written informed consent was waived due to the retrospective design. Patients aged 18–70 undergoing elective cardiac surgery with CPB from December 2018 to April 2021 were enrolled in China. The models were developed using the data from Fuwai Hospital and externally validated by the other three cardiac centres. Traditional logistic regression (LR) and eleven ML models were constructed. The primary outcome was PMI, defined as the postoperative maximum cardiac Troponin I beyond different times of upper reference limit (40x, 70x, 100x, 130x) We measured the model performance by examining the area under the receiver operating characteristic curve (AUROC), precision-recall curve (AUPRC), and calibration brier score.

**Results:**

A total of 2983 eligible patients eventually participated in both the model development (*n* = 2420) and external validation (*n* = 563). The CatboostClassifier and RandomForestClassifier emerged as potential alternatives to the LR model for predicting PMI. The AUROC demonstrated an increase with each of the four cutoffs, peaking at 100x URL in the testing dataset and at 70x URL in the external validation dataset. However, it’s worth noting that the AUPRC decreased with each cutoff increment. Additionally, the Brier loss score decreased as the cutoffs increased, reaching its lowest point at 0.16 with a 130x URL cutoff. Moreover, extended CPB time, aortic duration, elevated preoperative N-terminal brain sodium peptide, reduced preoperative neutrophil count, higher body mass index, and increased high-sensitivity C-reactive protein levels were identified as risk factors for PMI across all four cutoff values.

**Conclusions:**

The CatboostClassifier and RandomForestClassifer algorithms could be an alternative for LR in prediction of PMI. Furthermore, preoperative higher N-terminal brain sodium peptide and lower high-sensitivity C-reactive protein were strong risk factor for PMI, the underlying mechanism require further investigation.

**Supplementary Information:**

The online version contains supplementary material available at 10.1186/s13019-024-02856-y.

## Introduction

Annually, between 1 and 1.25 million cardiac surgeries are performed worldwide [1]; However, cardio-surgical procedures may induce flow disturbances during cardiopulmonary bypass (CPB), which can lead to perioperative myocardial injury (PMI) [2, 3]. Additionally, temporary ischemic episodes, cardioplegia reperfusion, and varying vasopressor and inotrope doses can exacerbate myocardial damage [4]. Myocardial injury leads to the release of specific biomarkers like cardiac troponin (cTn) I and T, as well as creatine kinase myocardial band (CK-MB). Elevated cTn levels above the 99th percentile upper reference limit (URL) indicate PMI [5].The recommendations on the optimal cut-off of the available biomarkers for the PMI differ significantly as the great variation of the biomarker’s kinetics and assay kits [5, 6]. The PMI with different cutoff values affects the prognosis [7, 8]. However, there is no relevant research on how cutoff values affect the risk prediction ability.

Machine learning (ML), with its thriving in the medicine domain, has been validated as an efficacious data preprocessing approach [9–12]. However, the performance of ML predicting PMI remains unknown.

Hence, in this study, we hypothesized that ML models, alongside traditional logistic regression, would demonstrate effective performance in estimating the risk of PMI using patient-specific variables across various cardiovascular surgical types involving CPB. Additionally, we expected that the performance evaluation would be conducted using four cardiac centers in China and considering four different cTn cutoff values (40x, 70x, 100x, 130x URL).

## Materials & methods

### Study design and participants

**Fig. 1:**
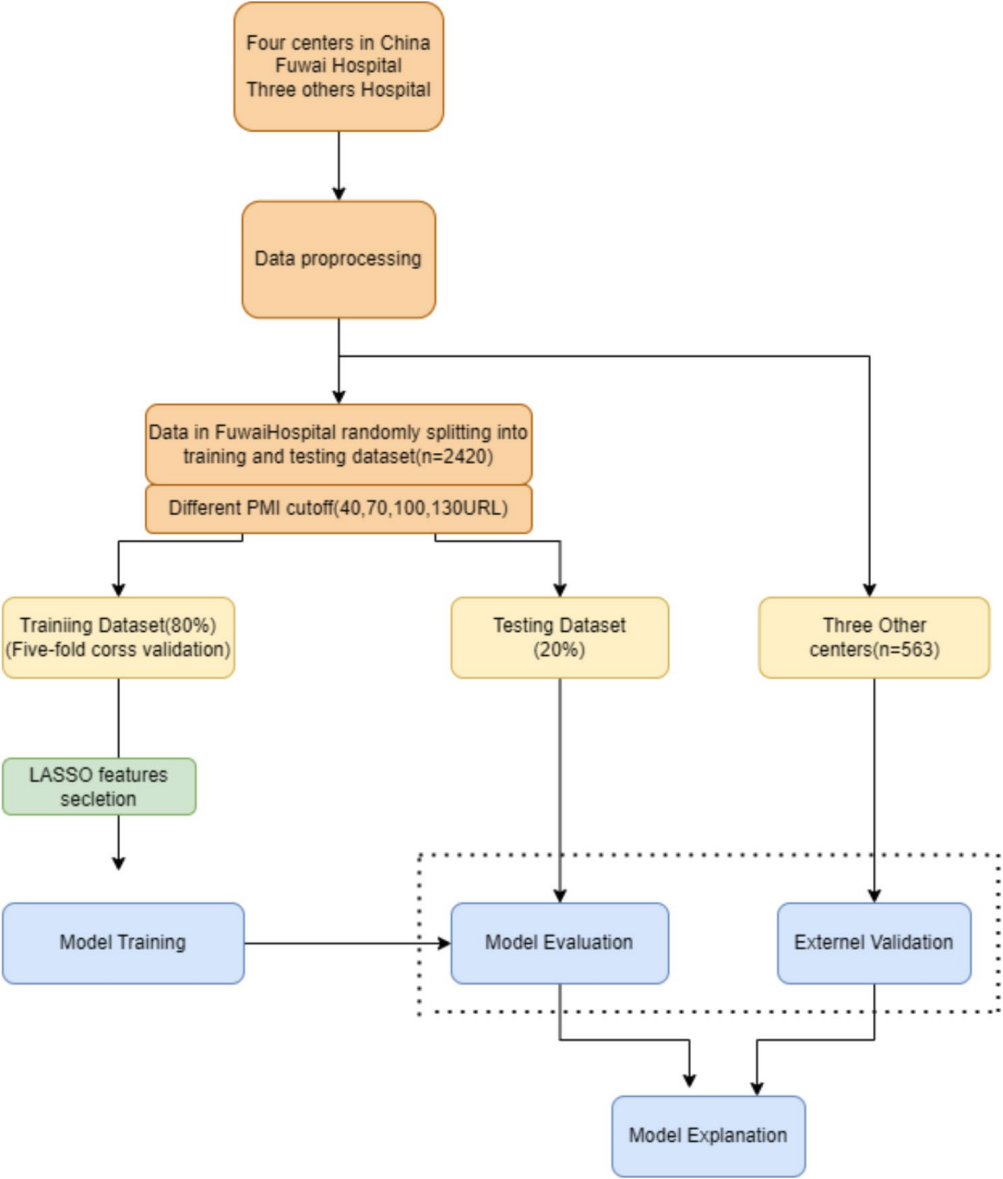
The overall review of this study

This study was a second analysis based on a multi-center randomized clinical trial (OPTIMAL, conducted at four cardiac centers in China) [13], approved by the Ethics Committee of Fuwai Hospital in Beijing (2018 − 1055) and the requirement for written informed consent was waived due to the retrospective design. An overview of the experimental design is presented in Fig. 1.

Patient inclusion criteria were as follows: (1) male or female adult patients aged 18-70 years, (2) patients who underwent elective surgery with CPB at our institution from December 2018 to April 2021. Patients without a record of cTnI were excluded. Preoperative and intraoperative variables, including demographic characteristics, baseline laboratory values, medical history, medication history, surgery time, CPB time, aortic clamp time, and surgery type, were extracted.

The present study adheres to the applicable Transparent Reporting of a multivariable prediction model for Individual Prognosis Or Diagnosis (TRIPOD) guidelines [14].

### PMI definition

The primary outcome was PMI, defined as the postoperative peak cTnI beyond different times of URL (40x, 70x, 100x, 130x) [7, 8].

### Model development and evaluation

The enrolled data of Fuwai Hospital (Beijing) were randomly assigned to 80% for the training dataset and 20% for the testing dataset. Furthermore, the data collected from three other cardiac centres in China were utilized for external validation (Fuwai Yunnan Cardiovascular Hospital, the First Affiliated Hospital of Wenzhou Medical University, and Fuwai Central China Cardiovascular Hospital). Development and validation datasets were imputed separately with mean values for continuous variables and frequency for categorical variables. In addition, the standard scaler data normalization technique was utilized to convert the data. A total of sixty available variables were captured to construct the predictive models. Features were selected in the training dataset using the least absolute shrinkage and selection operator (LASSO). In the LASSO model, the coefficients of variables were shrunk to zero, which means they were eliminated from the model.

Data were trained on the following models: (1) LR, set as the benchmark of the traditional method, (2) support vector machine (SVM), (3) KNeighborsClassifier (KNN), (4) Naive Bayes (BAY), (5) decision tree (DT), (6) RandomForest Classifier (RF), (7) Gradient Boosting Classifier (GB), (8) XGBoosting Classifier (XGB), (9) Light Gradient Boosting Machine (LGBM), (10) CatBoost Classifier (CAT), (11) AdaBoostClassifier, (12) ExtraTreeClassifier. Furthermore, a grid search with a five-fold cross-validation was performed on the training dataset to optimize hyperparameters.

The area under the receiver operating characteristic curve (AUROC) and the precision-recall (AUPRC) curve were utilized to discriminate the models. The brier score and calibration curve were executed to demonstrate the model calibration. Meanwhile, the accuracy, precision, recall score, and F1 score were also performed to evaluate the model comprehensively. Furthermore, decision curve analysis was also conducted. We selected the best-performing model based on the combination of these three metrics in the following order of priority: the highest AUROC, AUPRC, and well calibration curve. In addition, visualization of all features was performed, along with ranked feature importance, as derived from the SHapley Additive exPlanations (SHAP) interpreter [15].The methods were in accordance with our previous published paper [16].

### Statistical analysis

Categorical variables were presented in numbers and percentages, and continuous variables were presented as mean with standard deviation (SD) or median (Q1, Q3). The normality test was conducted on the continuous variables. The χ2 test and Fischer’s exact test were used for categorical variables (*p* < 0.05 indicates statistical significance). The student’s t-test and the Mann–Whitney U test were applied for continuous variables.

Python programming language (Python Software Foundation, version 3.9.7 and integrated development environment Jupyter Notebook 1.1.0) and SPSS software version 26.0 (IBM Corp., Armonk, New York, USA) were applied in our analysis.

## Results

### Patient characteristics

A total of 2983 eligible patients were eventually included in this study, 2420 of whom were from Fuwai Hospital (Beijing) for model development (1936 for the training and 484 for the testing dataset), and 563 from three other cardiac centres were for external validation. Four different cut-off values of cTn (40x, 70x, 100x, 130x) were used to define PMI. The demographics of the development and validation dataset are described in Table 1.

**Table 1:** Demographics of development and ex-validation dataset

	All Dataset (*n* = 2983)	Development Dataset(*n* = 2420)	Ex-validation Dataset (*n* = 563)	*p*
Sex (n, %)				0.014
Male	1841 (61.7%)	1519 (62.8%)	322 (57.2%)	
Female	1142 (38.3%)	901 (37.2%)	241 (42.8%)	
Age (y), median (IQR)	55.1 (16.4)	54.7 (16.3)	56.3 (15.2)	< 0.001
BMI (kg/m2),	24.2(4.7)	24.3(4.5)	23.5(5.2)	< 0.001
Race				< 0.001
Race-1	2812 (94.3%)	2271 (93.8%)	541 (96.1%)	
Race-2	171 (5.7%)	149 (6.2%)	22 (3.9%)	
NYHA				< 0.001
NYHA-I	327 (11.0%)	289 (11.9%)	38 (6.7%)	
NYHA-II	1419 (47.6%)	1272 (52.6%)	147 (26.1%)	
NYHA-III	1036 (34.7%)	819 (33.8%)	217 (38.5%)	
NYAH-IV	201 (6.7%)	40 (1.7%)	161 (28.6%)	
Medical history (n, %)				
Diabetes	327 (11.0%)	259 (10.7%)	68 (12.1%)	0.347
CHD	762 (25.5%)	663 (27.4%)	99 (17.6%)	< 0.001
Aortic disease	308 (10.3%)	271 (11.2%)	37 (6.6%)	< 0.001
Valvular disease	2078 (69.7%)	1725 (71.3%)	353 (62.7%)	< 0.001
Congenital heart disease	475 (15.9%)	413 (17.1%)	62 (11.0%)	< 0.001
previous myocardial injury	190 (6.4%)	163(6.7%)	27 (4.8%)	0.09
PVD	425(14.2%)	392(16.2%)	33(5.9%)	< 0.001
Smoke	1010 (33.9%)	832 (34.4%)	178 (31.6%)	0.212
Smoke1m	437 (14.6%)	329 (13.6%)	108 (19.2%)	< 0.001
Allergy	259 (8.7%)	229 (9.5%)	30 (5.3%)	0.002
β-Blockers	1217 (40.8%)	928 (38.3%)	289 (51.3%)	< 0.001
Statin	286 (9.6%)	210 (8.7%)	76 (13.5%)	< 0.001
ACEI	227 (7.6%)	179 (7.4%)	48 (8.5%)	0.363
Hyperlipidemia	989 (33.2%)	874 (36.1%)	115 (20.4%)	< 0.001
Hypertension	1022 (34.3%)	803 (33.2%)	219 (38.9%)	0.01
COPD	8 (0.3%)	4 (0.2%)	4 (0.7%)	0.046
CKD	11 (0.4%)	5 (0.2%)	6 (1.1%)	0.002
Infective endocarditis	30 (1.0%)	18 (0.7%)	12 (2.1%)	0.003
Non-invasive tests suggesting carotid artery stenosis > 79% or Stroke	161(5.4%)	109 (4.5%)	52 (9.2%)	< 0.001
Previous cardiac surgery	180(6.1%)	147(6.1%)	33(5.9%)	0.012
Previous carotid surgery	19 (0.6%)	14 (0.6%)	5 (0.9%)	0.406
Vital signs				
Body Temperature, °C	36.6(0.4)	36.4(0.3)	36.5(0.5)	
Heart rate, bpm/min	76.0 (17.0)	77.0 (18.0)	75.0 (17.0)	< 0.001
SD (mm Hg)	51(23)	52(23)	44(20)	< 0.001
LVEF (%)	61.0 (5.0)	61.0 (5.0)	61.0 (8.0)	0.025
LVEDD (mm)	51.0 (13.0)	51.0 (13.0)	53.0 (13.5)	0.004
Laboratory results				
WBC, ×10 L	6.1 (2.1)	6.0 (2.0)	6.2 (2.4)	0.005
Neutrophils, ×10 L	68.1 (12.8)	69.3 (11.5)	61.0 (13.3)	< 0.001
Hemoglobin	137.0 (22.0)	138.0 (22.0)	133.0 (23.0)	< 0.001
Platelets, × 10/L	200.0 (75.0)	200.0 (73.0)	199.0 (79.0)	0.973
AST (U/L)	25.0 (11.0)	25.0 (10.0)	21.0 (11.0)	< 0.001
AL T (U/L)	19.0 (16.5)	19.0 (16.0)	19.0 (18.0)	0.205
ALP (U/L)	67.0 (24.0)	65.0 (24.0)	76.5 (22.5)	< 0.001
GGT (U/L)	26.0 (24.7)	25.5 (22.0)	31.0 (23.7)	< 0.001
Direct bilirubin (µmol/L)	3.5 (2.9)	3.3 (2.5)	5.0 (3.0)	< 0.001
Total bilirubin, (µmol/L)	12.0 (7.3)	11.9 (7.3)	12.0 (7.9)	0.34
Baseline creatine mg/DL)	80.0 (22.0)	82.0 (21.2)	71.0 (22.5)	< 0.001
BUN (mg/DL)	5.9 (2.5)	6.0 (2.5)	5.8 (2.5)	0.008
TP (mg/DL)	67.6 (7.2)	67.7 (7.2)	67.1 (7.8)	0.002
PT, s	13.1 (1.0)	13.1 (1.0)	12.1 (2.5)	< 0.001
ALB (mg/DL)	40.0 (4.4)	39.8 (4.2)	40.3 (4.7)	< 0.001
NT-pro BNP (LN), (pg/ml)	266.9 (706.0)	276.5 (711.9)	235.0 (688.5)	0.342
D-Dimer	0.2 (0.2)	0.2 (0.2)	0.3 (0.4)	< 0.001
H’s-CRP (mg/DL)	1.1 (2.8)	0.8 (1.9)	4.5 (6.1)	< 0.001
Surgical information				
Lowest CPB-temperature, °C	32.0(1.8)	32.0(1.9)	31.8(1.2)	
CPB-HGB, g/DL,	102.0 (19.0)	102.5 (19.0)	99.0 (19.0)	< 0.001
Emergency, (%)	56(1.9%)	23(1.0%)	33(5.9%)	< 0.001
Dose of TxA, mg/Kg	62.7 (79.3)	60.8 (76.4)	73.8 (99.0)	< 0.001
Surgery type, n (%)				
isolated Valvular	1908 (64.0%)	1521 (62.9%)	387 (68.7%)	0.009
isolated CABG	824 (27.6%)	719 (29.7%)	105 (18.7%)	< 0.001
isolated Congenital	402 (13.5%)	342 (14.1%)	60 (10.7%)	0.05
isolated Aortic	216 (7.2%)	187 (7.7%)	29 (5.2%)	0.034
Surgery time(min)	253.0 (110.0)	241.0 (91.0)	340.0 (145.0)	< 0.001
CPB time (min)	121.0 (68.0)	114.0 (61.0)	159.0 (78.0)	< 0.001
Aorta clamp time (min)	84.0 (55.0)	81.0 (51.0)	105.0 (71.0)	< 0.001

Abbreviation: BMI: Body Mass Index; Race-1: Han-Chinese; Race-2: Chinese except for Han; NYHA: Classification of New York Heart Association; CHD: Coronary Heart Disease; PVD: Peripheral Vascular Disease; ACEI: angiotensin-converting enzyme inhibitors; COPD: Chronic Obstructive Pulmonary Disease; CKD: Chronic Renal Dysfunction; SD: Pulse pressure difference; LVEF: Left Ventricular Ejection Fraction; LVEDD: Left Ventricular end-diastolic diameter; WBC: white blood cells; ALT: alanine aminotransferase, AST: alkaline phosphatase; ALP: Alkaline phosphatase GGT: glutamyl transpeptidase; BUN urea nitrogen; TP: total protein; PT: prothrombin time; ALB: Albumin; Nt-proBNP: In (N-terminal brain sodium peptide); Hs-CRP: High-sensitivity C-reactive protein; CPB- HGB: the hemoglobin at the end of cardiopulmonary bypass; CABG: cardiac artery bypass grafting; TxA: Tranexamic acid

### Model construction

We constructed the eleven ML algorithms and LR models based on the approaches mentioned above. The LASSO selected the following features enter the final models: preoperative variables including age; sex; body mass index (weight (kg)/ (height [m]2);heart rate; pulse pressure difference; left ventricular ejection fraction; left ventricular end diastolic dimension; medical history such as diabetes, hypertension, previous history of cardiovascular disease, history of infective myocardial, previous carotid surgery, stroke and stenosis; medications including β-blocker and statin; and laboratory test results including the count of white blood cells, neutrophil, hemoglobin, platelet, aspartate aminotransferase, alkaline phosphatase, serum creatine, blood urea nitrogen, total protein, serum albumin, prothrombin time, d-dimer, N-terminal-pro brain natriuretic peptide (NT-pro BNP), high-sensitivity C-reactive protein (Hs-CRP), Intraoperative variables including surgery, CPB and aortic time, haemoglobin at end of CPB, and surgical type.

### Model performance of different PMI cut-off values

**Fig. 2:**
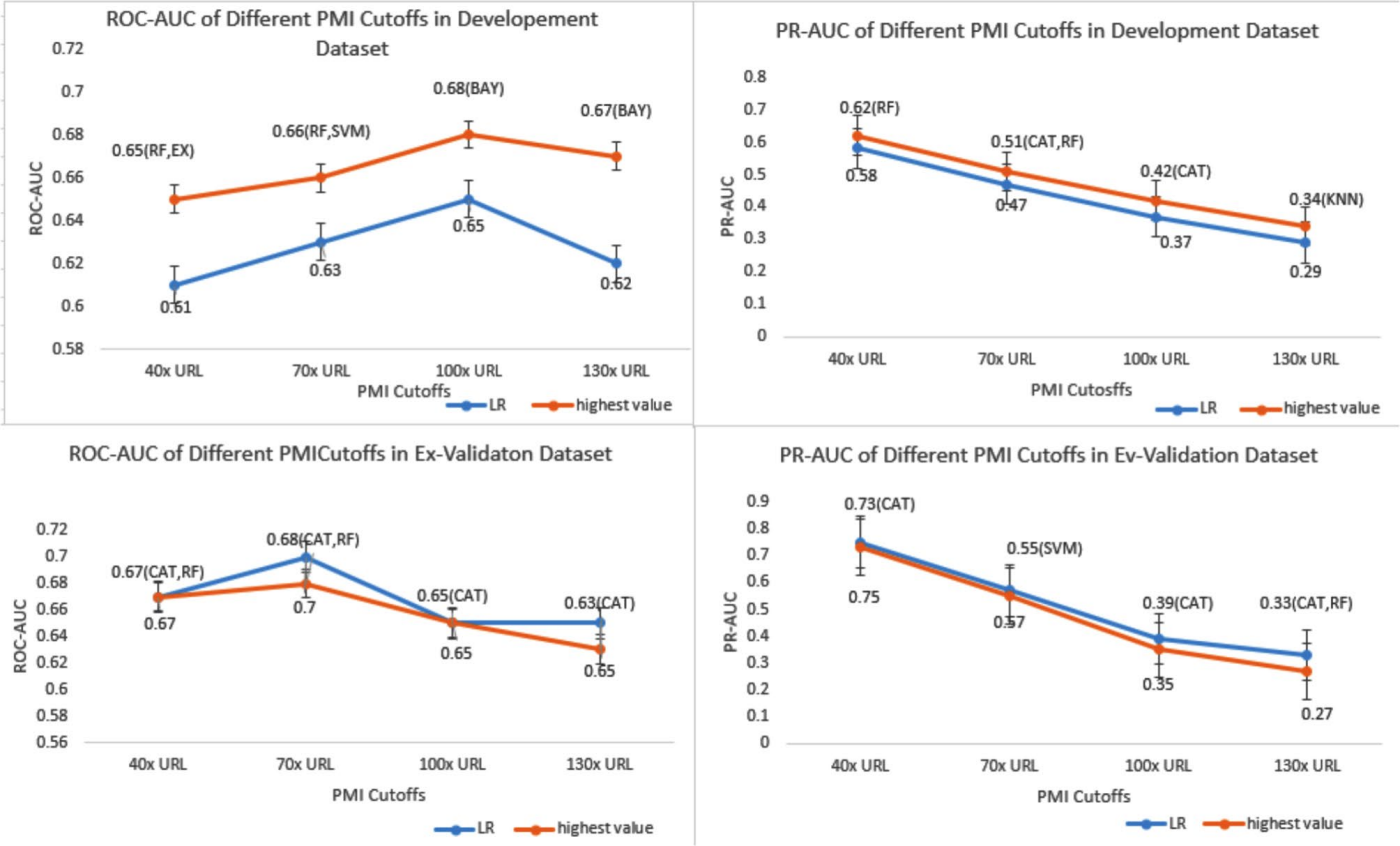
(**A**) The ROC-AUC of logistic regression and the highest value of AUROC among the elven machine learning algorithms with 40x,70x,100x,130x URL in the development dataset, (**B**) The ROC-AUC of logistic regression and the highest value of AUROC among the elven machine learning algorithms with 40x,70x,100x,130x URL in the external validation dataset, (**C**) The PR-AUC of logistic regression and the highest value of AUPRC among the elven machine learning algorithms with 40x,70x,100x,130x URL in the development dataset, (**D**) The PR-AUC of logistic regression and the highest value of AUPRC among the elven machine learning algorithms with 40x,70x,100x,130x URL in the external validation dataset. SVM: support vector machine; AB: AdaBoostClassifier; RF: RamdomForestClassifier; CAT: CatboostClassifier; EX: ExtraTreeClassifier; LGBM: LGBMClassifier; GB: GradientBoostingClassifier

The model performances with different PMI cutoff values were calculated across twelve ML algorithms. The AUC with varying cutoffs were summarized in Fig. 2.

In the testing dataset, LR achieved AUROCs of 0.61,0.63,0.65 and 0.62, with corresponding AUPRCs of 0.58,0.47,0.37,0.29, and Brier score of 0.24,0.22,0.19,0.16 for cutoff 40x,70x,100x and 130x URL, respectively. Moreover, among the eleven ML algorithms, the highest AUROCs were 0.65,0.66,0.68 and 0.67,the highest AUPRCs were 0.62,0.51,0.42, and 0.32, and the lowest Brier score were 0.24, 0.22,0.18, and 0.16 for the same cutoff values.

In the external validation dataset, the LR model achieved AUPRCs of 0.67,0.70,0.65, and 0.65, with corresponding AUPRCs of 0.75,0.57,0.39, and 0.33,and Brier score were 0.23,0.21,0.19, and 0.17 for cutoff 40x,70x,100x and 130x URL, respectively. Additionally, among the other eleven ML algorithms, the highest AUROCs were 0.67,0.68,0.65 and 0.63, the highest AUPRCs were 0.73,0.55,0.35, and 0.27 ,the lowest Brier scores were 0.24, 0.21,0.18, and 0.16 for the same cutoff values. Detailed performance of different PMI cut-offs is shown in Supplementary Figs. 1–3, supplementary Tables 1–4.

**Fig. 3:**
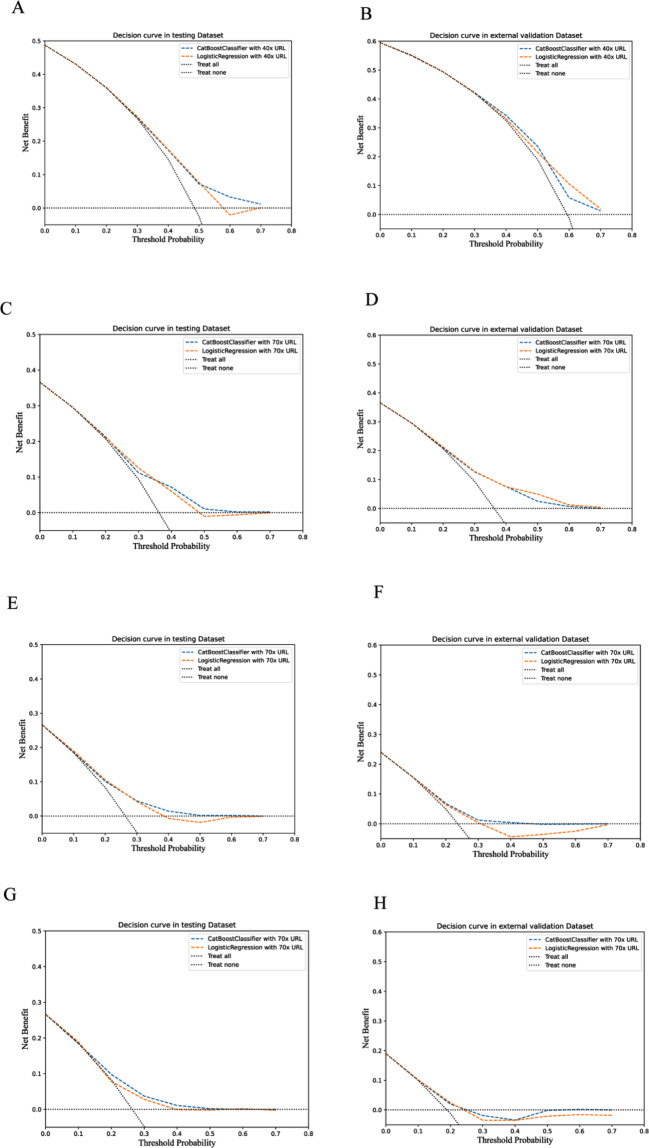
(**A**)The decision curves for 40x URL PMI in the testing dataset, (**B**) the decision curves for 40x URL PMI in the external validation dataset, (**C**)The decision curves for 70x URL PMI in the testing dataset, (**D**) the decision curves for 70x URL PMI in the external validation dataset, (**E**)The decision curves for 100x URL PMI in the testing dataset, (**F**) the decision curves for 100x URL PMI in the external validation dataset, (**G**)The decision curves for 130x URL PMI in the testing dataset, (**H**) the decision curves for 130x URL PMI in the external validation dataset

Furthermore, the decision curves with four PMI cutoffs are presented in Fig. 3.

### SHAP interpreter for the models

SHapley Additive exPlanations (SHAP) summary plot was applied to illustrate the feature importance of the predictive model. High SHAP values indicate an increased risk of PMI. According to the CAT classifier model, in the testing dataset, the top five features with a 40x URL were coronary artery bypass graft (CABG) surgery type, Hs-CRP, body temperature, hemoglobin of end CPB, and neutrophil count; the top five features with a 70x URL were CABG surgery, NT-pro BNP, CPB time, aortic time, and Hs-CRP; the top five with a 100x URL were CPB time, aortic time, NT-pro BNP, surgery time and Hs-CRP; and the top five with a 130x URL were CPB time, aortic time, NT-pro BNP, surgery time and CABG surgery.

In the external validation dataset, the top five features with a 40x URL were Hs-CRP, CABG surgery type, neutrophil count, body temperature, and prothrombin time; the top five features with a 70x URL were CPB time, NT-pro BNP, CABG surgery, aortic time and surgery time; the top five with a 100x URL were CPB time, aortic time, surgery time, Hs-CRP and NT-pro BNP; the top five with a 130x URL were CPB time, aortic time, surgery time NT-pro BNP and Hs-CRP. The SHAP values of different cutoffs are presented in the Supplementary Fig. 4.

## Discussion

In this retrospective cohort study, we have developed and externally validated the model performance using eleven ML models and the traditional LR method based on four different PMI cutoffs. Consequently, the ML models, especially the CAT and RF models, exhibited better performance in the discrimination and calibration compared to LR model, showing a potential alternative for LR. Additionally, the top five risk factors across all four cutoffs were prolonged CPB, aortic duration, surgery time, elevated preoperative Nt-proBNP, and decreased preoperative Hs-CRP. These findings highlight the potential use of CAT and RF models in estimating PMI risk and guiding clinical decision-making in cardiac surgery.

To our knowledge, this is the first study to focus on establishing the ML predictive model for PMI with a large sample size. In this study, with various cardiovascular types and URL cutoffs, the CAT model showed potential candidates for forecasting PMI risk among the eleven ML algorithms. The CAT algorithm, a binary recursive segmentation technology, could yield convincing results with limited training data and computational power by reducing the calculating time, overfitting chances, and tuning the hyperparameter burden [17, 18].

PMI, a common complication after cardiac surgery, has been identified to be associated with substantial short and long-term mortality [19–21]. However, the cTnI values between different assays and manufacturers may influence the cutoff of PMI, and most of the studies mainly focused on CABG and percutaneous coronary intervention(PCI) [22, 23]. Thus, we have investigated a wide range of cardiovascular types for the potential risk of PMI. Moreover, we have explored the predictive model with a wide range of cut-off URLs for PMI [6–8]. The AUROC exhibited an upward trend with each of the four cutoffs, reaching its peak at 100x URL in the testing dataset and at 70x URL in the external validation dataset. However, it’s important to note that the AUPRC decreased with each increment in cutoff. Furthermore, the Brier loss score decreased as the cutoffs increased, reaching its lowest point at 0.16 with a 130x URL cutoff.

Furthermore, we have explored the potential risk factors for PMI with four different cutoffs. The previous study has reported that preoperative high-dose statin loading played an essential role in preventing PMI by downregulating the release kinetics of cardiac biomarkers such as cTnI, CK-MB, and Nt-proBNP [24]. In addition, hypotension and transcription orchestration played a crucial role in PMI [25, 26]. However, the above studies merely analyzed the potential perioperative risk factor for PMI during cardiac surgery with CPB. In this study, CPB and aortic clamp time were in a strongly positive correlation with PMI. The plausible reasons may attribute to the following two folds. First, the activation of systematic inflammation response mediated by the CPB circuit upregulates inflammatory cytokines and small molecules such as interleukins-8, interleukins-10, and tumor necrosis factor α, which could exacerbate myocardial injury [27]. More importantly, a longer duration of CPB is associated with the increase of plasma levels of soluble syndecan-1, a signal for endothelial glycocalyx degradation, which could precipitate neutrophil egress from the bone marrow contributing to and dilating the systemic inflammatory response [28]. Furthermore, prolonged CPB time and aorta clamp time were significantly associated with endotoxin levels. The intestinal mucosa is especially vulnerable to hypoperfusion during CPB. The endotoxin could be dispersed into the circulating blood, exacerbating the myocardial injury [29]. It is desperate to enhance the patient management during cardiac surgery, reducing the incidence of severe complications, especially PMI, which is primarily clinically silent and only ascertained by routine troponin screening. Of note, over 90% of elevated troponin patients are absent in ischemia-related evidence of electrocardiographic or echocardiographic. Thus they could not be diagnosed as myocardial infarction defined in the 4th Universal Definition [5].

Consistent with a previous double-blind, randomized controlled trial, the higher Nt-proBNP levels could predict PMI following elective vascular operations [30]. Our study confirmed that the preoperative Nt-proBNP was positively correlated with PMI. Although the level of Nt-proBNP is the marker of the overload volume and is utilized to guide outpatient therapy among patients with heart failure [31], a previous study found an additional mechanism of its release, with the potential to modify oxidant stress in the heart [30]. Furthermore, we also confirmed that the valvular surgery type was more inclined to suffer PMI [32]. Intriguing, the preoperative neutrophils, BMI, and Hs-CRP were negative correlated with PMI, which needs further prospective investigation.

There are several limitations. First, our study was a retrospective design, which may accompany some immeasurable confounding biases. Although we conducted an external validation, further validation is needed before our models are affirmatively applied to other populations, institutions, and regions. Second, our risk model is tailored to patients undergoing cardiac surgery with CPB, which may be inapplicable in other surgery types. Third, the population in our study has mainly undergone valve surgery, which may limit the use in other surgery. Fourth, the optimal cutoff needs more extensive and detailed investigations.

## Conclusions

The CAT and RF algorithms could be an alternative for LR in prediction of PMI. Furthermore, preoperative higher Nt-proBNP and lower Hs-CRP were strong risk factor for PMI, the underlying mechanism require further investigation.

### Electronic supplementary material

Below is the link to the electronic supplementary material.

Supplementary Material 1

## Data Availability

No datasets were generated or analysed during the current study.
