# Supplementary material for: Development and validation of a machine learning predictive model for perioperative myocardial injury in cardiac surgery with cardiopulmonary bypass
Source: J Cardiothorac Surg. 2024 Jun 26;19:384. doi: 10.1186/s13019-024-02856-y (PMC11201784; doi:10.1186/s13019-024-02856-y)
Supplement: Supplementary file 1 — Supplementary Material 1 [file 13019_2024_2856_MOESM1_ESM.docx]

**Supplementary materials**

**Figure 1**:(A) The AUC of the machine learning models under the 40x URL cut-off in the testing dataset,(B) The AUC of the machine learning models under the 40x URL cut-off in the validation dataset,(C) The AUC of the machine learning models under the 70x URL cut-off in the testing dataset,(D) The AUC of the machine learning models under the 70x URL cut-off in the validation dataset,(E) The AUC of the machine learning models under the 100x URL cut-off in the testing dataset,(F) The AUC of the machine learning models under the 100x URL cut-off in the validation dataset,(G) The AUC of the machine learning models under the 130x URL cut-off in the testing dataset,(H) The AUC of the machine learning models under the 130x URL cut-off in the validation dataset.





**Figure2**: (A) The Precision recall curve of the machine learning models under the 40x URL cut-off in the testing dataset,(B) The Precision recall curve in the machine learning models under the 40x URL cut-off in the validation dataset,(C) The Precision recall curve of the machine learning models under the 70x URL cut-off in the testing dataset,(D) The Precision recall curve in the machine learning models under the 70x URL cut-off in the validation dataset,(E) The Precision recall curve of the machine learning models under the 100x URL cut-off in the testing dataset,(F) The Precision recall curve in the machine learning models under the 100x URL cut-off in the validation dataset,(G) The Precision recall curve of the machine learning models under the 130x URL cut-off in the testing dataset,(H) The Precision recall curve in the machine learning models under the 130x URL cut-off in the validation dataset.

**

**

**Figure 3**:(A) The Calibration curve of the machine learning models under the 40x URL cut-off in the testing dataset,(B) The Calibration curve in the machine learning models under the 40x URL cut-off in the validation dataset,(C) The Calibration curve of the machine learning models under the 70x URL cut-off in the testing dataset,(D) The Calibration curve in the machine learning models under the 70x URL cut-off in the validation dataset,(E) The Calibration curve of the machine learning models under the 100x URL cut-off in the testing dataset,(F) The Calibration curve in the machine learning models under the 100x URL cut-off in the validation dataset,(G) The Calibration curve of the machine learning models under the 130x URL cut-off in the testing dataset,(H) The Calibration curve in the machine learning models under the 130x URL cut-off in the validation dataset.

**Figure 4**: (A) The Top 20 features of the CatboostClassifier model under the 40x URL cut-off in the testing dataset by SHAP model explainer,(B)The Top 20 features of the CatboostClassifier model under the 40x URL cut-off in the validation dataset by SHAP model explainer,(C)The Top 20 features of the CatboostClassifier model under the 70x URL cut-off in the testing dataset by SHAP model explainer,(D) The Top 20 features of the CatboostClassifier model under the 70x URL cut-off in the validation dataset by SHAP model explainer,(E) The Top 20 features of the CatboostClassifier model under the 100x URL cut-off in the testing dataset by the model explainer,(F) The Top 20 features of the CatboostClassifier model under the 100x URL cut-off in the validation dataset by the model explainer, (G) The Top 20 features of the CatboostClassifier model under the 130x URL cut-off in the testing dataset by the model explainer,(H) The Top 20 features of the CatboostClassifier model under the 130x URL cut-off in the validation dataset by the model explainer

Abbreviation:

BMI: Body Mass Index; Race-1: Han-Chinese; Race-2: Chinese except for Han.

NYHA: Classification of New York Heart Association

CHD: Coronary Heart Disease; PVD: Peripheral Vascular Disease; ACEI: angiotensin-converting enzyme inhibitors; COPD: Chronic Obstructive Pulmonary Disease; CKD: Chronic Renal Dysfunction; SD: Pulse pressure difference; LVEF: Left Ventricular Ejection Fraction; LVEDD: Left Ventricular end-diastolic diameter; WBC: white blood cells; ALT: alanine aminotransferase, AST: alkaline phosphatase; ALP: Alkaline phosphatase ;GGT: glutamyl transpeptidase; BUN :urea nitrogen; TP: total protein; PT: prothrombin time; ALB: Albumin; NT-proBNP: In (N-terminal brain sodium peptide); Hs-CRP: High-sensitivity C-reactive protein;

CPB- HGB: the hemoglobin of cardiopulmonary bypass.

CABG: cardiac artery bypass grafting

**Table 1**: The overall evaluation metrics of the machine learning models under the 40x URL cut-off in the testing and external validation datasets.

|  | AUROC | | Brier loss | | Log loss | | Accuracy | | Precision | | Recall | | F1 | |
| --- | --- | --- | --- | --- | --- | --- | --- | --- | --- | --- | --- | --- | --- | --- |
| Classifier | Testing | Ex-Val | Testing | Ex-Val | Testing | Ex-Val | Testing | Ex-Val | Testing | Ex-Val | Testing | Ex-Val | Testing | Ex-Val |
| Logistic Regression | 0.61 | 0.67 | 0.24 | 0.23 | 0.67 | 0.65 | 0.59 | 0.62 | 0.58 | 0.68 | 0.60 | 0.69 | 0.59 | 0.68 |
| Support Vector Machine | 0.64 | 0.65 | 0.24 | 0.24 | 0.66 | 0.67 | 0.61 | 0.61 | 0.58 | 0.67 | 0.72 | 0.66 | 0.64 | 0.67 |
| KNeighborsClassifier | 0.62 | 0.60 | 0.24 | 0.24 | 0.67 | 0.67 | 0.61 | 0.60 | 0.60 | 0.65 | 0.61 | 0.69 | 0.61 | 0.67 |
| DecisionTreeClassifier | 0.53 | 0.53 | 0.48 | 0.47 | 17.13 | 16.97 | 0.52 | 0.53 | 0.51 | 0.62 | 0.56 | 0.53 | 0.54 | 0.57 |
| RandomForestClassifier | 0.65 | 0.67 | 0.24 | 0.24 | 0.67 | 0.67 | 0.60 | 0.64 | 0.57 | 0.70 | 0.69 | 0.70 | 0.63 | 0.70 |
| GaussianNB | 0.62 | 0.61 | 0.31 | 0.32 | 1.61 | 2.39 | 0.58 | 0.58 | 0.55 | 0.65 | 0.70 | 0.65 | 0.62 | 0.65 |
| GradientBoostingClassifier | 0.60 | 0.61 | 0.26 | 0.26 | 0.71 | 0.72 | 0.57 | 0.56 | 0.56 | 0.68 | 0.57 | 0.50 | 0.56 | 0.58 |
| XGBClassifier | 0.61 | 0.63 | 0.24 | 0.24 | 0.67 | 0.67 | 0.59 | 0.62 | 0.58 | 0.71 | 0.59 | 0.60 | 0.58 | 0.65 |
| LGBMClassifier | 0.60 | 0.63 | 0.24 | 0.24 | 0.68 | 0.67 | 0.57 | 0.62 | 0.56 | 0.69 | 0.57 | 0.65 | 0.56 | 0.67 |
| CatboostClassifier | 0.64 | 0.67 | 0.24 | 0.23 | 0.67 | 0.65 | 0.58 | 0.64 | 0.57 | 0.71 | 0.61 | 0.67 | 0.59 | 0.69 |
| AdaBoostClassifier | 0.62 | 0.63 | 0.25 | 0.25 | 0.69 | 0.68 | 0.58 | 0.62 | 0.56 | 0.67 | 0.61 | 0.70 | 0.59 | 0.68 |
| ExtraTreeClassifier | 0.65 | 0.63 | 0.24 | 0.24 | 0.66 | 0.66 | 0.60 | 0.60 | 0.57 | 0.63 | 0.74 | 0.76 | 0.64 | 0.69 |

**Table 2**: The overall evaluation metrics of the machine learning models under the 70x URL cut-off in the testing and external validation datasets.

|  | AUROC | | Brier loss | | Log loss | | Accuracy | | Precision | | Recall | | F1 | |
| --- | --- | --- | --- | --- | --- | --- | --- | --- | --- | --- | --- | --- | --- | --- |
| Classifier | Testing | Ex-Val | Testing | Ex-Val | Testing | Ex-Val | Testing | Ex-Val | Testing | Ex-Val | Testing | Ex-Val | Testing | Ex-Val |
| Logistic Regression | 0.63 | 0.70 | 0.22 | 0.21 | 0.63 | 0.61 | 0.62 | 0.68 | 0.46 | 0.62 | 0.18 | 0.35 | 0.26 | 0.45 |
| Support Vector Machine | 0.66 | 0.67 | 0.22 | 0.21 | 0.63 | 0.62 | 0.63 | 0.63 | 0.00 | 0.00 | 0.00 | 0.00 | 0.00 | 0.00 |
| KNeighborsClassifier | 0.62 | 0.63 | 0.22 | 0.22 | 0.64 | 0.64 | 0.65 | 0.63 | 0.55 | 0.50 | 0.23 | 0.26 | 0.32 | 0.34 |
| DecisionTreeClassifier | 0.54 | 0.53 | 0.43 | 0.46 | 15.64 | 16.52 | 0.57 | 0.54 | 0.41 | 0.40 | 0.42 | 0.48 | 0.42 | 0.43 |
| RandomForestClassifier | 0.66 | 0.68 | 0.22 | 0.22 | 0.63 | 0.63 | 0.64 | 0.64 | 0.59 | 0.61 | 0.09 | 0.07 | 0.16 | 0.12 |
| GaussianNB | 0.65 | 0.64 | 0.30 | 0.33 | 1.78 | 2.37 | 0.58 | 0.57 | 0.45 | 0.45 | 0.67 | 0.75 | 0.54 | 0.56 |
| GradientBoostingClassifier | 0.64 | 0.61 | 0.23 | 0.23 | 0.64 | 0.66 | 0.62 | 0.65 | 0.48 | 0.55 | 0.33 | 0.29 | 0.39 | 0.38 |
| XGBClassifier | 0.64 | 0.64 | 0.22 | 0.22 | 0.63 | 0.63 | 0.65 | 0.64 | 0.56 | 0.52 | 0.25 | 0.16 | 0.34 | 0.25 |
| LGBMClassifier | 0.64 | 0.65 | 0.22 | 0.22 | 0.63 | 0.63 | 0.64 | 0.67 | 0.55 | 0.69 | 0.16 | 0.17 | 0.25 | 0.28 |
| CatboostClassifier | 0.65 | 0.68 | 0.22 | 0.21 | 0.62 | 0.62 | 0.64 | 0.66 | 0.54 | 0.60 | 0.19 | 0.20 | 0.28 | 0.30 |
| AdaBoostClassifier | 0.66 | 0.65 | 0.24 | 0.24 | 0.67 | 0.67 | 0.63 | 0.65 | 0.47 | 0.63 | 0.05 | 0.11 | 0.09 | 0.18 |
| ExtraTreeClassifier | 0.66 | 0.65 | 0.22 | 0.22 | 0.63 | 0.63 | 0.64 | 0.67 | 0.53 | 0.71 | 0.11 | 0.16 | 0.19 | 0.25 |

**Table 3**: The overall evaluation metrics of the machine learning models under the 100x URL cut-off in the testing and external validation datasets.

|  | AUROC | | Brier loss | | Log loss | | Accuracy | | Precision | | Recall | | F1 | |
| --- | --- | --- | --- | --- | --- | --- | --- | --- | --- | --- | --- | --- | --- | --- |
| Classifier | Testing | Ex-Val | Testing | Ex-Val | Testing | Ex-Val | Testing | Ex-Val | Testing | Ex-Val | Testing | Ex-Val | Testing | Ex-Val |
| Logistic Regression | 0.65 | 0.65 | 0.19 | 0.19 | 0.56 | 0.56 | 0.71 | 0.72 | 0.38 | 0.40 | 0.11 | 0.31 | 0.17 | 0.35 |
| Support Vector Machine | 0.61 | 0.60 | 0.19 | 0.19 | 0.56 | 0.56 | 0.73 | 0.76 | 0.00 | 0.00 | 0.00 | 0.00 | 0.00 | 0.00 |
| KNeighborsClassifier | 0.60 | 0.59 | 0.19 | 0.19 | 0.57 | 0.63 | 0.74 | 0.73 | 0.56 | 0.31 | 0.07 | 0.12 | 0.12 | 0.17 |
| DecisionTreeClassifier | 0.55 | 0.52 | 0.37 | 0.42 | 13.48 | 15.11 | 0.63 | 0.58 | 0.32 | 0.26 | 0.37 | 0.39 | 0.35 | 0.31 |
| RandomForestClassifier | 0.66 | 0.59 | 0.19 | 0.19 | 0.56 | 0.56 | 0.73 | 0.76 | 0.00 | 0.00 | 0.00 | 0.00 | 0.00 | 0.00 |
| GaussianNB | 0.68 | 0.60 | 0.71 | 0.73 | 6.85 | 8.71 | 0.27 | 0.27 | 0.27 | 0.24 | 0.98 | 0.97 | 0.42 | 0.39 |
| GradientBoostingClassifier | 0.64 | 0.62 | 0.19 | 0.18 | 0.56 | 0.55 | 0.73 | 0.74 | 0.47 | 0.35 | 0.07 | 0.10 | 0.12 | 0.16 |
| XGBClassifier | 0.65 | 0.63 | 0.19 | 0.18 | 0.56 | 0.54 | 0.73 | 0.76 | 0.00 | 0.57 | 0.00 | 0.03 | 0.00 | 0.06 |
| LGBMClassifier | 0.62 | 0.63 | 0.19 | 0.18 | 0.56 | 0.54 | 0.74 | 0.76 | 0.53 | 0.48 | 0.07 | 0.10 | 0.12 | 0.16 |
| CatboostClassifier | 0.66 | 0.65 | 0.18 | 0.18 | 0.55 | 0.54 | 0.74 | 0.76 | 0.55 | 0.47 | 0.05 | 0.05 | 0.09 | 0.09 |
| AdaBoostClassifier | 0.65 | 0.57 | 0.22 | 0.22 | 0.63 | 0.63 | 0.73 | 0.76 | 0.00 | 1.00 | 0.00 | 0.01 | 0.00 | 0.01 |
| ExtraTreeClassifier | 0.66 | 0.61 | 0.19 | 0.18 | 0.55 | 0.55 | 0.73 | 0.76 | 0.00 | 0.67 | 0.00 | 0.01 | 0.00 | 0.03 |

**Table 4**: The overall evaluation metrics of the machine learning models under the 130x URL cut-off in the testing and external validation datasets.

|  | AUROC | | Brier loss | | Log loss | | Accuracy | | Precision | | Recall | | F1 | |
| --- | --- | --- | --- | --- | --- | --- | --- | --- | --- | --- | --- | --- | --- | --- |
| Classifier | Testing | Ex-Val | Testing | Ex-Val | Testing | Ex-Val | Testing | Ex-Val | Testing | Ex-Val | Testing | Ex-Val | Testing | Ex-Val |
| Logistic Regression | 0.62 | 0.65 | 0.16 | 0.17 | 0.50 | 0.51 | 0.79 | 0.79 | 0.41 | 0.41 | 0.07 | 0.26 | 0.12 | 0.32 |
| Support Vector Machine | 0.60 | 0.55 | 0.17 | 0.18 | 0.51 | 0.71 | 0.79 | 0.77 | 0.00 | 0.16 | 0.00 | 0.05 | 0.00 | 0.07 |
| KNeighborsClassifier | 0.61 | 0.58 | 0.16 | 0.16 | 0.50 | 0.57 | 0.80 | 0.80 | 1.00 | 0.21 | 0.04 | 0.03 | 0.08 | 0.05 |
| DecisionTreeClassifier | 0.54 | 0.55 | 0.30 | 0.38 | 10.87 | 13.57 | 0.70 | 0.62 | 0.27 | 0.24 | 0.26 | 0.44 | 0.26 | 0.31 |
| RandomForestClassifier | 0.65 | 0.60 | 0.16 | 0.16 | 0.49 | 0.50 | 0.79 | 0.81 | 0.00 | 0.00 | 0.00 | 0.00 | 0.00 | 0.00 |
| GaussianNB | 0.67 | 0.56 | 0.74 | 0.79 | 7.20 | 10.18 | 0.24 | 0.21 | 0.21 | 0.19 | 0.98 | 0.97 | 0.35 | 0.32 |
| GradientBoostingClassifier | 0.62 | 0.61 | 0.16 | 0.16 | 0.51 | 0.50 | 0.78 | 0.79 | 0.31 | 0.34 | 0.05 | 0.14 | 0.09 | 0.20 |
| XGBClassifier | 0.65 | 0.63 | 0.16 | 0.15 | 0.49 | 0.48 | 0.79 | 0.79 | 0.44 | 0.13 | 0.04 | 0.02 | 0.07 | 0.03 |
| LGBMClassifier | 0.62 | 0.61 | 0.16 | 0.16 | 0.51 | 0.49 | 0.79 | 0.79 | 0.38 | 0.11 | 0.06 | 0.02 | 0.10 | 0.03 |
| CatboostClassifier | 0.65 | 0.63 | 0.16 | 0.16 | 0.49 | 0.49 | 0.80 | 0.81 | 1.00 | 0.43 | 0.01 | 0.03 | 0.02 | 0.05 |
| AdaBoostClassifier | 0.65 | 0.54 | 0.20 | 0.21 | 0.59 | 0.60 | 0.79 | 0.81 | 0.00 | 0.00 | 0.00 | 0.00 | 0.00 | 0.00 |
| ExtraTreeClassifier | 0.66 | 0.57 | 0.16 | 0.16 | 0.49 | 0.50 | 0.79 | 0.81 | 0.00 | 0.50 | 0.00 | 0.01 | 0.00 | 0.02 |

**Supplementary Table 5**: The final enrolled variables and the missing percent in the development and validation datasets.

|  | Development Dataset | Ex-validation Dataset |
| --- | --- | --- |
|  | n=2420 | n=563 |
|  |  |  |
| Sex (n, %) | 0.00% | 0.00% |
| Male |  |  |
| Female |  |  |
| Age (y), median (Q1, Q3) | 0.00% | 0.00% |
| Body Mass Index (kg/m2), | 0.00% | 2.13% |
| Left Ventricular Ejection Fraction | 0.04% | 3.91% |
| Left Ventricular End Diastolic Dimension | 3.76% | 4.44% |
| Medical history (n, %) |  |  |
| Diabetes | 0.00% | 0.00% |
| smoke | 0.04% | 0.00% |
| Valvular disease | 0.17% | 0.18% |
| Peripheral Vascular Disease | 0.17% | 0.18% |
| Hypertension | 3.47% | 0.00% |
| Congenital heart disease | 0.17% | 0.18% |
| Coronary Heart disease | 0.17% | 0.18% |
| Aortic disease | 0.17% | 0.18% |
| Infective endocarditis | 0.08% | 0.18% |
| Non-invasive tests suggesting carotid artery stenosis >79% or Stroke | 0.00% | 0.00% |
| Previous cardiac surgery | 3.76% | 0.00% |
| Previous carotid surgery | 4.55% | 0.18% |
| β-Blockers | 1.78% | 0.00% |
| Statin | 1.78% | 0.00% |
| Vital signs |  |  |
| Body Temperature, °C | 0.00% | 0.00% |
| Heart rate, bpm/min | 0.00% | 0.00% |
| Pulse pressure (mm Hg) | 0.21% | 0.00% |
| Laboratory results |  |  |
| white blood cells, ×10/L | 0.00% | 0.71% |
| Neutrophils, ×10L | 0.54% |  |
| Haemoglobin, g/DL, | 0.00% | 0.71% |
| Platelets, × 10/L | 0.00% | 0.71% |
| alkaline phosphatase (U/L) | 0.08% | 2.13% |
| alkaline phosphatase (U/L) | 0.41% | 15.45% |
| Prothrombin Time | 0.33% | 0.36% |
| Baseline creatinine, (mg/DL) | 0.00% | 0.36% |
| alkaline phosphatase (mg/DL) | 0.04% | 1.60% |
| Albumin (mg/DL) | 0.04% | 3.73% |
| Total Protein | 0.04% | 3.73% |
| NT-pro BNP (pg/ml) | 4.96% | 1.95% |
| D-Dimer | 1.40% | 3.37% |
| Hs-CRP (mg/DL) | 0.37% | 6.57% |
| Surgery type, n (%) |  |  |
| Valvular surgery | 0.17% | 0.18% |
| Coronary Artery Bypass Graft | 0.17% | 0.18% |
| Congenital heart disease surgery | 0.17% | 0.18% |
| Aortic surgery | 0.17% |  |
| Surgery time(min) | 0.00% | 1.78% |
| CPB time (min) | 0.37% | 1.07% |
| Aorta clamp time (min) | 0.99% | 1.78% |
| Hemoglobin at the end of CPB, g/DL, | 2.48% | 2.66% |

Abbreviation: NT-pro BNP: N-terminal-pro brain natriuretic peptide ;Hs-CRP: high-sensitivity C-reactive protein
